# Angiolymphoid hyperplasia with eosinophilia: a case report 

**DOI:** 10.1186/s13256-018-1599-x

**Published:** 2018-04-02

**Authors:** Alexey Youssef, Ali Ramez Hasan, Youssef Youssef, Lina Al-soufi, Yahya Elshimali, Zuheir Alshehabi

**Affiliations:** 1 Faculty of Medicine, Tishreen University, Lattakia, Syria; 2 Department of Otolaryngology and Head and Neck Surgery, Tishreen University Hospital, Tishreen University, Lattakia, Syria; 3 Ministry of Health, National Hospital of Lattakia, Lattakia, Syria; 4 Department of Pathology, University of California Los Angeles, Charles Drew University, Los Angeles, California USA; 5 Department of Pathology, Tishreen University Hospital, Tishreen University, Lattakia, Syria

**Keywords:** Angiolymphoid hyperplasia with eosinophilia, Therapeutic approach, Pediatric disorders, Peculiar presentation

## Abstract

**Background:**

Angiolymphoid hyperplasia with eosinophilia is a benign neoplasm that includes blood vessel proliferation and a dense eosinophilic inflammatory infiltrate. Mostly, it affects middle-aged adults manifesting as flesh/plum-colored pruritic nodules and papules, most commonly affecting the ear and the periauricular area.

**Case presentation:**

In this case, we report a 13-year-old Caucasian girl with bilateral, huge, protruding, and yellowish nostril masses which were peculiar in location and of gross appearance. At first, the disease proved to be a diagnostic dilemma. After making a diagnosis of angiolymphoid hyperplasia with eosinophilia, the disease also proved to be a therapeutic dilemma. It did not respond to oral prednisolone or to oral indomethacin, and it proved to be resistant to topical steroids. Although surgery is the standard therapeutic approach, it recurred despite multiple surgical attempts. However, the only regimen that seemed to partially control the lesion was intralesional steroids combined with topical tacrolimus ointment.

**Conclusions:**

Angiolymphoid hyperplasia with eosinophilia proves a therapeutic dilemma, because there is a large variety of proposed treatments, yet there is not enough data on most of them. Although the disease is not deadly by itself, it usually presents with disfiguring lesions that grimly affect the patient’s quality of life. This warrants further research and efforts to find an effective cure and a unified therapeutic approach.

## Background

Angiolymphoid hyperplasia with eosinophilia (ALHE) is a benign neoplasm of debated etiology. Most commonly, it affects the face and the periauricular area especially, where it usually manifests as papules or nodules. We describe here a distinctive case with regard to the location and gross appearance of the tumor.

## Case presentation

**Fig. 1:**
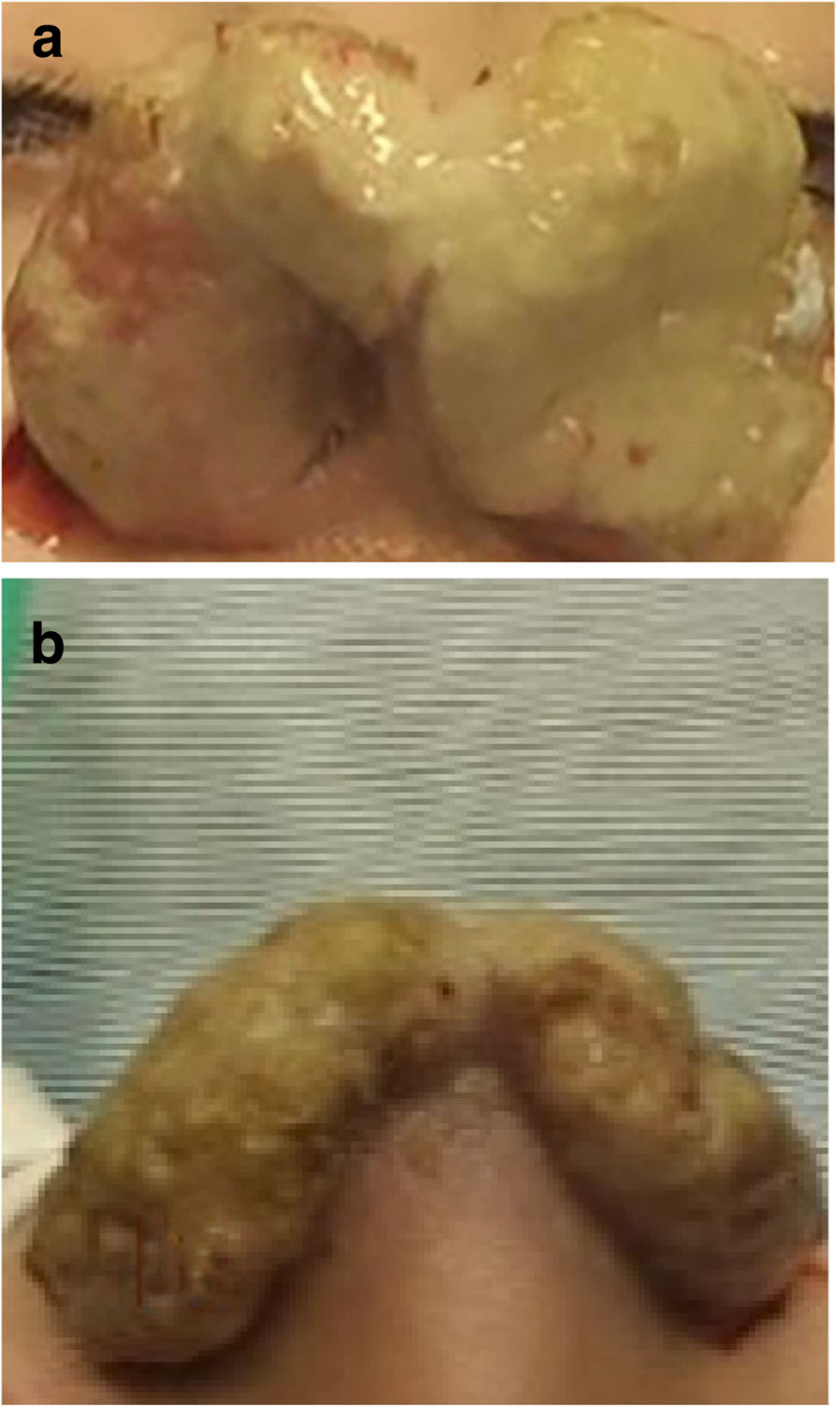
Presurgical photograph. The mass appears as a congested and yellowish tumor, protruding outside the nostrils. **a** caudal view of the lesion. **b**. superior view of the lesion

**Fig. 2:**
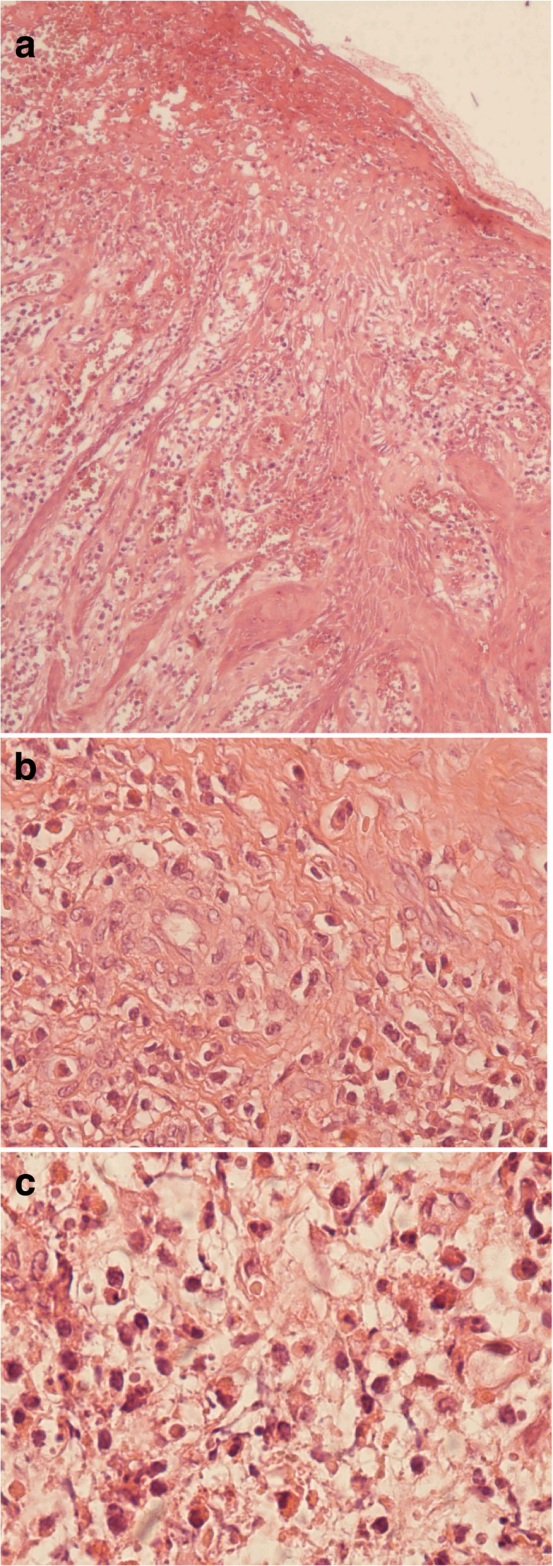
Histopathologic study and description. **a** Skin biopsy, nose - pseudoepitheliomatous hyperplasia with ulceration and necrosis. Diffuse lymphohistiocytic infiltrate with numerous eosinophils in a fibrous stroma (hematoxylin and eosin, × 40). **b** Skin biopsy, nose - vascular proliferation with prominent (plump) endothelial cells and occasional cytoplasmic vacuoles (hematoxylin and eosin, × 60). **c** Skin biopsy, nose - vascular proliferation with intense eosinophilic infiltrate (hematoxylin and eosin, × 100)

**Fig. 3:**
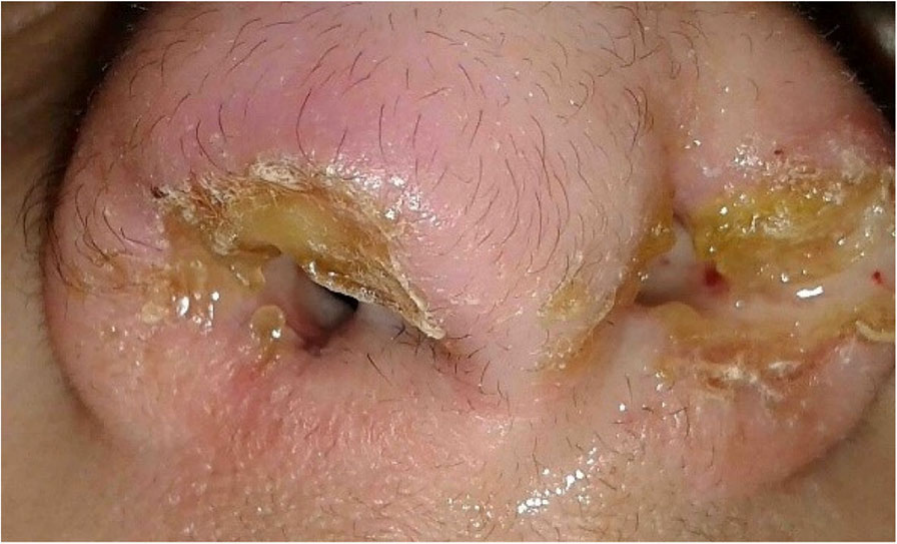
The results of postsurgical treatment with intralesional steroids and tacrolimus cream

**Fig. 4:**
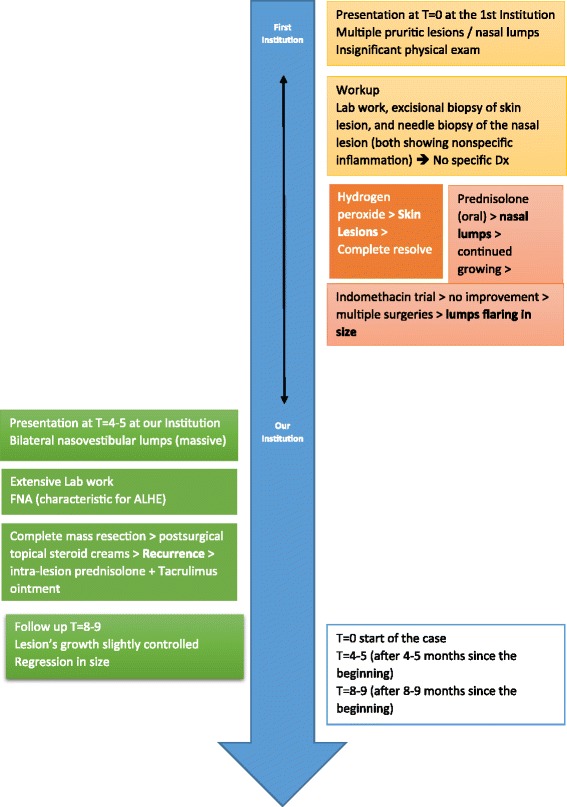
Treatment of angiolymphoid hyperplasia with eosinophilia

Our 13-year-old patient is a Caucasian girl with an unremarkable medical history, yet her family history is significant for allergies and asthma. She initially presented, to another institute, with multiple pruritic facial skin lesions and a pruritic left intranasal lump. Apart from having two erythematous pruritic plaques in the left suborbital region and a yellowish pruritic lump occupying the left nasal vestibule, her physical examination proved to be insignificant without any lymphadenopathies or salivary gland enlargements. Consequently, a laboratory workup was conducted in addition to an excisional biopsy of one dermatologic lesion and a needle biopsy of the nasal lesion. Both biopsies exhibited nonspecific inflammation with granulation, necrosis, and no signs of malignancy. No specific diagnosis was made. The patient was started on hydrogen peroxide treatment for the skin lesions, which resolved completely with no recurrence. Simultaneously, another lump started growing in the right nasal vestibule. Suspecting an inflammatory etiology, she was started on oral prednisolone 1 mg/kg/day by mouth twice a day. Despite therapy, these nasal lumps continued growing. Hence, prednisolone was discontinued after 10 days of therapy to start an indomethacin trial of 1.5 mg/kg/day by mouth twice a day for 14 days, which was also discontinued due to its inefficacy. Then a decision to perform surgery was taken, and the presurgical computed tomography (CT) scan revealed bilateral soft tissue masses arising from the right and left nasal vestibules. Although she had undergone many surgical attempts to remove the lumps, none of them succeeded and both lumps flared in size. After a couple of months, our patient presented to our institution with bilateral nasovestibular lumps; they were massive in size, occluding nasal entrance and protruding outside the nose (Fig. 1). We did an extensive laboratory workup to exclude any comorbidities (Table 1). We did an fine-needle aspiration (FNA) biopsy of the lesion, which was diagnostic of ALHE (Fig. 2). Our following surgical attempt included complete mass resection. Despite surgery and postsurgical treatment with topical steroid creams, the lesion recurred. Consequently, we started the patient on intralesional prednisolone twice a month and topical 0.1% tacrolimus ointment twice daily. This latter regimen seemed to slightly control the lesion’s growth, causing a limited regression in size after 4 months of treatment (Fig. 3). A timeline of the patient's case can be seen in (Fig. 4).

**Table 1:** Laboratory values throughout the disease course

	Lab results at the other institution	Lab results at time of diagnosis	4 months after surgery	Units	Reference range
Leukocytes	11,700	12,000	8000	C/uL	3500–10,000
Neutrophils	72	68	60	%	34–71
Eosinophils	**15.2**	**16**	5	%	0.7–6
Lymphocytes	8	10	25	%	19–51
Erythrocytes	4.80	4.70	5	×103/mm^3^	M 4.5–6.2 F 4–5.4
Hemoglobin	14	14	15	g/dL	M 13–18 F 12–16
CRP	3.2	4	1.5	mg/L	0.5–5
ESR	35	45	14	mm/hL	Up to 15
Tuberculin test		Negative			Negative
ANCA c		2.5 Negative		U	Positive more than 18 Negative less than 18
ANCA p		2.2 Negative		U	Positive more than 18 Less than 18
HHV8:					
IgG		8.1 Positive		U	Positive more than 1.1
IgM		0.2 Negative		U	Negative less than 0.9
IgE	1997	1200	250	IU/mL	U to 120
Creatinine	0.6	0.7	0.6	mg/dL	0.5–1.2
Urea	25	23	20	mg/dL	5–45
ALT		6		U/L	5–40
AST		18		U/L	0–40
Urine protein		Negative	Negative		Negative
Urine casts		0	0	Number/field	0
Blood film		Within normal			

*CRP* C-reactive protein, *ESR* erythrocytes sedimentation rate, *ANCA p* antineutrophil cytoplasmic antibodies perinuclear, *ANCA c* antineutrophil cytoplasmic antibodies cytoplasmic, *HHV8* human herpes virus 8, *IgG* immunoglobulin G, *IgM* immunoglobulin M, *IgE* immunoglobulin E, *ALT* alanine transferase, *AST* aspartate transferase. We typed the eosinophils numbers in bold to highlight their abnormal value

Our patient’s parents reported her full adherence to treatment. The patient herself reported decreased quality of life and impaired social interactions due to the disfiguring lesions. She also reported marked fear and distress because of the ineffectiveness of multiple therapeutic regimens and surgeries. The patient’s family reported severe financial burden due to the high costs of the treatments.

## Discussion

The term angiolymphoid hyperplasia with eosinophilia (ALHE) was first created (in 1969) by Wells and Whimster [1], who used it to describe a distinguished neoplasm. The tumor is characterized by a florid proliferation of blood vessels lined by plump endothelial cells and admixed with a dense inflammatory infiltrate of lymphocytes, eosinophils, and mast cells. They reported nine cases in which the neoplasm remained benign although they reported recurrence after excision in some cases. Later, Weiss and Enzinger argued the nature of that entity, for they wanted to evidently differentiate the lesion from the malignant vascular tumor, epithelioid hemangioendothelioma. For this, they introduced the term epithelioid hemangioma (EH) in 1982 [1]. Since then, many hypotheses have been proposed to explain the etiology of the tumor, including a reactive process, a neoplastic process, and infectious mechanisms with possible association with HIV [1]. However, the argument that ALHE/EH may represent a monoclonal T-cell process is supported in some cases [2]. Interestingly, peripheral T-cell lymphoma has been reported to develop in a patient with ALHE/EH. Additionally, a certain association between ALHE/EH and follicular mucinosis has been reported. There are also cases of ALHE/EH in which T-cell receptor gene (TCR) rearrangement and monoclonality have been detected [1].

In attempt to define the histogenesis of this disorder, a team analyzed both the phenotype and the genotype of the inflammatory infiltrate using immunohistochemistry and TCR gene rearrangement by polymerase chain reaction (PCR) and other methods. The results showed five out of seven patients with ALHE displaying a clonal T-cell population and proliferative T-cell activity in ALHE tissue; most of these cases were recurrent following a prolonged and therapy-resistant course. Unfortunately, these tests were not available for our case because of war restrictions [2].

In 2015, a statistical analysis yielded no sex predominance among 908 patients [3]. Over half of the patients presented with a single lesion, and most common locations were the ear, and periauricular area; face; and scalp. Furthermore, the analysis revealed a consequential association between the existence of multiple lesions and pruritus along with bleeding. Considering age, statistics has shown a wide prevalence range (0.7 months to 91 years) and the mean age of presentation was 37.6 years. According to the literature [3], cases of ALHE with earlier age of onset, longer duration, and multiple lesions were linked to higher recurrence rates after excision, which exactly fits the course of our case. Although the ear is the most common location, in our case the lesion was located in the nostrils. To the best of our knowledge, only one similar case of ALHE in the nostrils has been reported in the medical literature [4]. While ALHE usually present as flesh- to plum-colored papules or nodules, our patient had bilateral soft tissue masses of congestive nature and a distinctive yellowish color. Kharkwal *et al*. have reported a similar unusual gross appearance [5].

ALHE is commonly associated with neither an elevated level of immunoglobulin E (IgE) nor an eosinophilia; in contrast, these two signs are common findings in Kiruma’s disease [1]. Eosinophilia is evident in only 20% of ALHE cases and elevated IgE levels is quite a rare finding [2]. Unexpectedly, our patient had both signs (remarkably high IgE level and eosinophilia). While Kimura’s disease surmounts the differential diagnosis list, it also includes angiomatous lymphoid hamartoma, hemangioma, pyogenic granuloma, Kaposi sarcoma, lymphoma, and epithelioid hemangioendothelioma [1].

**Fig. 5:**
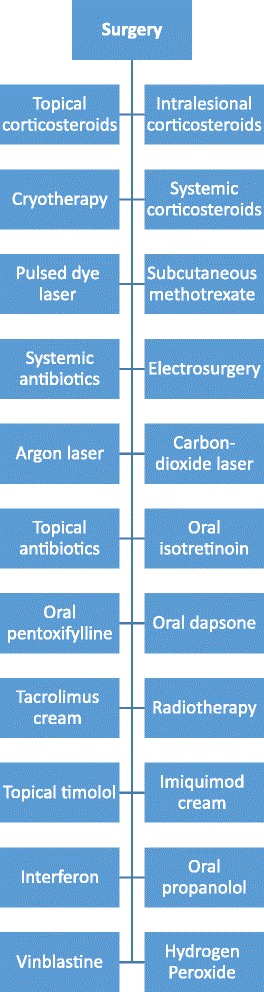
A list of the available treatment options for ALHE in addition to hydrogen pyroxide which was effective in our case

Considering the highly recurring nature of ALHE, there is no single best therapeutic approach. Surgery is the most commonly used one (with a 40% recurrence rate), followed by intralesional and topical corticosteroids. In our case, our final approach was intralesional corticosteroids and topical tacrolimus cream, which eventually proved a slight effectiveness. Here, we provide a figure describing the available treatment options in addition to hydrogen peroxide, which was effective for the suborbital lesions in our case (Fig. 5) [3, 4, 6–8].

## Conclusions

What is peculiar about our case is the gross appearance and unconventional location of the intranasal lesions and the high levels of IgE and eosinophilia, which is rather uncommon in ALHE. Taking into account the highly debilitating and disfiguring nature of the disease, more efforts should be aimed towards creating a standardized and effective therapeutic approach that could help physicians treat such a recurring disease.

## Abbreviations

ALHE: Angiolymphoid hyperplasia with eosinophilia
ALT: Alanine transferase
ANA: Antinuclear antibodies
ANCA c: Antineutrophil cytoplasmic antibodies cytoplasmic
ANCA p: antineutrophil cytoplasmic antibodies perinuclear
AST: Aspartate transferase
CRP: C-reactive protein
EH: Epithelioid hemangioma
ESR: Erythrocytes sedimentation rate
FNA: Fine-needle aspiration
HHV8: Human herpes virus 8
IgE: Immunoglobulin E
IgG: Immunoglobulin G
IgM: Immunoglobulin M
PCR: Polymerase chain reaction
TCR: T-cell receptor gene

## Authors’ information

AY is a medical student. ARH is a medical student. YY is a professor of otolaryngology. LS is a dermatologist. ZA is a professor of pathology. YE is a professor of pathology/oncology.
